# Association Between Initiation, Intensity, and Cessation of Smoking and Mortality Risk in Patients With Cardiovascular Disease: A Cohort Study

**DOI:** 10.3389/fcvm.2021.728217

**Published:** 2021-12-15

**Authors:** Jiang-lin Wang, Wen-jun Yin, Ling-yun Zhou, Ya-feng Wang, Xiao-cong Zuo

**Affiliations:** ^1^Department of Pharmacy, The Third Xiangya Hospital, Central South University, Changsha, China; ^2^Department of Epidemiology and Biostatistics, School of Health Sciences, Wuhan University, Wuhan, China; ^3^Department of Pharmacy and Center of Clinical Pharmacology, The Third Xiangya Hospital, Central South University, Changsha, China

**Keywords:** cardiovascular disease, smoking status, all-cause mortality, cardiovascular disease mortality, cancer mortality

## Abstract

**Objectives:** To examine the effect of smoking status, smoking intensity, duration of smoking cessation and age of smoking initiation on the risk of all-cause and cause-specific mortality among cardiovascular disease (CVD) patients.

**Design:** A population-based prospective cohort study.

**Setting:** The National Health Interview Survey (NHIS) in the U.S. that were linked to the National Death Index (NDI).

**Participants:** 66,190 CVD participants ≥ 18 years of age who were interviewed between 1997 and 2013 in the NHIS linked to the NDI through December 31, 2015.

**Outcome Measures:** The primary outcome was all-cause mortality and the secondary outcome was cause-specific mortality including CVD mortality and cancer mortality.

**Results:** During the mean follow-up of 8.1 years, we documented 22,518 deaths (including 6,473 CVD deaths and 4,050 cancer deaths). In the overall CVD population, former and current smokers had higher risk of all-cause (Former smokers: hazard ratios (HRs), 1.26; 95% confidence interval (CI), 1.21–1.31, *P* < 0.001; Current smokers: HRs, 1.96; 95%CI, 1.86–2.07, *P* < 0.001), CVD (Former smokers: HRs, 1.12; 95%CI, 1.05–1.21, *P* = 0.001; Current smokers: HRs, 1.80; 95%CI, 1.64–1.97, *P* < 0.001) and cancer mortality (Former smokers: HRs, 1.49; 95%CI, 1.35–1.64, *P* < 0.001; Current smokers: HRs, 2.78; 95%CI, 2.49–3.09, *P* < 0.001) than never smokers. Furthermore, similar results were observed when the study subjects were stratified according to the type of CVD. Among current smokers, the risk for cancer mortality increased as the daily number of cigarettes increased, regardless of the specific type of CVD. However, the association of the risk for all-cause and CVD mortality with smoking intensity did not present a dose-response relationship. In participants with angina pectoris or stroke, smoking intensity was inversely associated with deaths from CVD. In addition, the risk for all-cause, CVD and cancer mortality declined as years of smoking cessation increased. Finally, the relative risk of all-cause mortality was not significantly different in individuals with a younger age of smoking initiation.

**Conclusions:** CVD patients who are smokers have an increased risk of all-cause, CVD and cancer mortality, and the risk decreases significantly after quitting smoking. These data further provide strong evidence that supports the recommendation to quit smoking for the prevention of premature deaths among individuals with CVD.

## Strengths and Limitations of This Study

➢ The key strengths of this study include its prospective design, large sample size, long-term follow-up and a large number of deaths, which allowed the investigation of long-term survival and provided sufficient deaths for analyses of more detailed causes of death, and careful adjustments for a multitude of potential risk factors.
➢ Additionally, unlike previous studies that only included subjects with specific CVD diseases, we included the entire CVD population and examined the association of all-cause and cause-specific mortality risk with smoking status, the age of smoking initiation, the number of cigarettes smoked per day and years since quitting.
➢ The CVD diseases, smoking status were self-reported and these information were self-reported only at baseline.
➢ The information on what types of tobacco products were smoked were not available.
➢ Since the NHIS is a passive follow-up study, the cause of death may be misclassified and the estimated hazards of cause-specific mortality may have been affected.

## Introduction

Cardiovascular disease (CVD) is the leading cause of mortality and has become a global public health crisis. The Global Burden of Disease (GBD) 2015 study estimated that 422.7 million individuals worldwide were affected by CVD in 2015 (1). Based on the most recent estimates from the GBD study, nearly 18 million individuals died from CVD, representing one-third of deaths globally (1, 2).

Smoking is recognized as a well-established major risk factor for CVD and the second leading attributable risk factor for death (3, 4). Even smokers who quit smoking for 10~15 years still have a higher risk of CVD than non-smokers (5). Although compelling evidence has indicated that smoking has harmful effects on health, approximately 25.0% of men and 5.4% of women are still daily smokers worldwide (6). In particular, a sizeable proportion (10 to 50%) of patients continued to smoke even after the diagnosis of CVD (7–10).

To date, the adverse influence of smoking on the risk of CVD (such as ischaemic heart disease, acute myocardial infarction, cerebrovascular disease, peripheral arterial disease, heart failure or other heart condition/disease) (11, 12) and all-cause and specific-cause mortality have been extensively studied and analyzed in the general population (13–16). However, among individuals with CVD, who have a 2- to 4-times higher risk of mortality than the general population, evidence regarding the association of smoking status with health outcomes remains inconclusive and insufficient. Previous studies have revealed a phenomenon called the “smoker's paradox”, implying that smokers who have developed myocardial infarction (MI), acute ischaemic stroke and acute heart failure had lower rates of adverse short-term outcomes, especially in-hospital mortality (17–19). Conversely, increasing evidence has recently indicated that smoking increases long-term adverse outcomes in patients with established CVD (7, 8, 20). However, the relationship between smoking status and adverse long-term outcomes in most of the above studies has so far been concentrated on patients with coronary artery disease, with limited evidence on the relation of smoking with long-term adverse outcomes among patients with different types of CVD. In addition, few studies have assessed the association of the age of smoking initiation, the number of cigarettes smoked per day and the duration since smoking cessation with long-term all-cause and cause-specific mortality, especially CVD and cancer mortality, among patients with established CVD.

Therefore, additional investigations of the associations between smoking and outcomes in CVD patients are warranted. The primary purpose of this study was to examine the effect of smoking status, smoking intensity, duration of smoking cessation and age of smoking initiation on the risk of all-cause mortality among CVD patients. The association between smoking status, smoking intensity, duration of smoking cessation, age of smoking initiation and cause-specific mortality was evaluated among CVD patients as a secondary objective. The broader purpose was to provide direct evidence for clinical practice and behavior changes.

## Methods

### Data Source

This study was conducted using data from the National Health Interview Survey (NHIS), which has been conducted continuously since 1957. The NHIS is a nationally representative annual cross-sectional household survey administered by the National Center for Health Statistics (NCHS) and uses a stratified, multistage sampling design to collect information from the civilian, non-institutionalized population of the United States. The content and structure of the NHIS has been revised approximately every 10–15 years. Two major revisions were made to the NHIS questionnaire in 1982 and 1997. To ensure the consistency of the self-reported information of the survey participants, we used data after 1997. Additional information about the NHIS has been described previously (21) and is provided by the Centers for Disease Control and Prevention (http://www.cdc.gov/nchs/nhis/about_nhis.htm). Adult participants ≥ 18 years of age from the annual 1997 to 2013 waves of the NHIS were included in the study (22, 23). Of these, 67,811 individuals with CVD were eligible to be linked to the National Death Index through December 31, 2015 (21). In addition, participants were excluded if they had incomplete information on smoking status and CVD types at baseline. After exclusion, 66,190 subjects were included in the final analysis. In this study, only public-use NHIS data were used. The public-use NHIS data are de-identified and do not include any protected health information. Our study was based on secondary analysis of public-use data. The publicly available data are considered as exempt under the ethical board review of the corresponding author's institution (the Third Xiangya Hospital of Central South University).

### Definition of CVD

Individuals were identified as having CVD based on positive responses to the following question: Have you ever been told by a doctor or other health professional that you had coronary heart disease (CHD), a heart attack (also MI), angina (also called angina pectoris), a stroke, or any kind of heart condition or heart disease?

### Assessment of Smoking

NHIS study participants were administered questionnaires relating to their smoking status, the age of smoking initiation, the number of cigarettes smoked per day, years since quitting and the number of cigarettes smoked per day before quitting. Based on self-reported responses for these questionnaires, survey participants were categorized into 3 categories for analysis: never smokers, former smokers, and current smokers. To analyse the effect of the age of smoking initiation and smoking intensity on outcomes, we classified current and former smokers into categories of age at smoking initiation: ≤ 12, 13–17, and ≥ 18+ years and categories of ≤ 9, 10–19 and ≥ 20 cigarettes smoked per day. In addition, we divided former smokers into categories of years since cessation: 0 to <10, 10 to <20 and ≥ 20 years in the smoking cessation analysis.

### Outcomes

The primary outcome was all-cause mortality. The secondary outcomes included CVD mortality (codes I00-I09, I11, I13, and I20-I51, I60-I69) and cancer mortality (codes C00-C97), according to the International Statistical Classification of Diseases, Injuries, and Causes of Death (ICD-9 for deaths prior to 1999 and ICD-10 for deaths occurring from 1999 onwards).

### Covariates

Several baseline variables were included as confounders in this study. Demographic data on age at enrolment, self-reported sex, self-reported race (Hispanic, non-Hispanic white, non-Hispanic black and non-Hispanic others), educational attainment (did not complete high school, completed high school, education beyond high school and missing education information) were collected. The following lifestyle behavior information was also included: alcohol consumption (lifetime abstainer, former drinker, current drinker, missing alcohol consumption information); physical activity, which was grouped into meeting recommendation, not meeting recommendation and missing data groups based on the 2008 Physical Activity Guidelines for Americans (24); and body mass index (BMI), which was calculated as weight in kilograms divided by height in meters squared and was categorized as underweight and normal weight (BMI, <25 kg/m^2^), overweight (BMI, 25 to <30 kg/m^2^) and obesity (BMI, ≥ 30 kg/m^2^). Socioeconomic status included household income, which was divided into low [poverty-to-income ratio (PIR), ≤ 1], moderate (PIR, 1 to <4) and high (PIR, ≥ 4) groups based on the family PIR (25). We also included self-reported physician-diagnosed medical comorbidities (diabetes, hypertension, cancer). Missing values were set to a separate missing data category for that particular covariate and were included as an indicator variable in the analysis.

### Statistical Analysis

Continuous variables are presented as the means with standard deviations (SEs), and categorical variables are presented as proportions. All the analyses performed were pre-specified in an analysis protocol, prior to the conduction of the study. First, for the analysis of smoking status, participants were categorized into non-smokers, former smokers, current smokers, and non-smokers, which was used as the reference group. We used Cox proportional hazards models to estimate hazard ratios (HRs) and 95% confidence intervals (CIs) for the association between smoking status and mortality. In the minimally adjusted model, age, sex, and race were adjusted. We additionally adjusted for education, income, BMI, physical activity, alcohol intake, baseline hypertension, diabetes and cancer variables in a multivariable-adjusted model. Second, we used Cox proportional hazards models to calculate HRs and 95% CIs for all-cause, CVD and cancer mortality in association with smoking intensity and the years of smoking cessation compared with the parameters in non-smokers. To further evaluate whether the age of smoking initiation was associated with all-cause mortality, we compared all-cause mortality in former and current smokers who started smoking at 12 or younger than 12 years and 13 to 17 years with those who started smoking at 18 or older than 18 years (reference group). Subsequently, subgroup analysis by five major CVDs, namely, CHD, stroke, MI, angina pectoris and other heart disease also was performed. Finally, we conducted a sensitivity analysis excluding the first 2 years of follow-up and excluding patients with cancer at baseline to test the robustness of our primary findings. The results should be interpreted as exploratory because this study did not account for multiple comparisons. For all analyses, survey weights were applied to account for the complex sampling design. All analyses were performed with Stata 13.0 statistical software (Stata Corp LP, College Station, TX, USA). Two-sided statistical tests were used, and a *P* value of less than 0.05 was considered an indication of statistical significance.

### Patient and Public Involvement

There was no patients and public involvement in the development, design or analysis of this study.

## Results

### Characteristics of Study Population

This study population consisted of 66,190 patients with CVD, of whom 32.6, 19.6, 25.6, 18.8 and 57.9% were diagnosed with CHD, stroke, MI, angina pectoris and other heart disease, respectively. Table 1 shows the participants' characteristics, subdivided by their self-reported smoking status. Among these participants, 50.8, 79.9 and 55.2% were women and non-Hispanic White and had a moderate household income, respectively. Overall, approximately 44.0% (29,668) did not smoke, 36.5% (23,439) were former smokers, and 19.6% were current smokers. There was a strong association between smoking status and alcohol drinking status. Namely, never smokers were more likely to be lifetime abstainers. However, current smokers were more likely to be current alcohol drinkers. Compared with current smokers, never and former smokers had higher education levels. In addition, physical activity was similar but less prevalent across all 3 categories of smoking status.

**Table 1:** Baseline demographic characteristics of CVD patients, according to smoking status.

**Characteristics**	**Never smoker**	**Former smoker**	**Current smoker**
No. (%)	29,668 (44.0)	23,439 (36.5)	13,083 (19.6)
**Age, mean (years)**	60.04 (0.2)	65.4 (0.1)	51.8 (0.2)
**Sex (%)**			
Female	20,011 (62.7)	9,954 (38.0)	6,756 (48.2)
**Race (%)**			
Hispanic	3,713 (8.8)	1,756 (5.2)	1,086 (5.8)
Non-Hispanic White	20,344 (75.8)	18,548 (85.0)	9,400 (79.6)
Non-Hispanic Black	4,523 (11.7)	2,608 (7.6)	2,252 (12.0)
Non-Hispanic Other	1, 088 (3.6)	527 (2.2)	345 (2.5)
**Education level (%)**			
Less than high school degree	7,453 (20.6)	6,055 (23.1)	3,758 (26.6)
High school degree	8,148 (28.2)	6,808 (29.8)	4,308 (34.9)
More than high school degree	13,830 (50.4)	10,450 (46.5)	4,938 (37.9)
Missing	237 (0.8)	126 (0.6)	79 (0.6)
**Income (%)**			
Low	5,494 (13.5)	3,099 (9.8)	3,684 (22.4)
Moderate	16,330 (53.6)	13,745 (56.8)	7,064 (55.8)
High	7,844 (32.9)	6,595 (33.4)	2,335 (21.8)
**BMI (kg/m** ^**2**^ **)**			
<25 (%)	10,191 (34.0)	6,917 (28.5)	5,221 (39.0)
25-30 (%)	9,703 (32.9)	8,517 (37.2)	4,176 (32.4)
>30 (%)	8,868 (30.1)	7,503 (32.2)	3,432 (26.6)
Missing	906 (3.0)	502 (2.1)	254 (1.9)
**Alcohol intake (%)**			
Lifetime abstainer	11,560 (36.3)	2,968 (11.6)	1,612 (11.7)
Former drinker	5,895 (19.1)	8,368 (34.2)	3,553 (26.6)
Current drinker	11,946 (43.8)	11,900 (53.4)	7,752 (60.4)
Missing	267 (0.9)	203 (0.8)	166 (1.3)
**Physical activity (%)**			
No meeting	20,206 (65.0)	6,917 (28.5)	5,221 (39.0)
Meeting	8,847 (32.8)	8,517 (37.2)	4,176 (32.4)
Missing	615 (2.2)	572 (2.6)	326 (2.8)
**Physician-diagnosed disease (%)**			
Hypertension	17,750 (56.6)	15,335 (63.9)	7,245 (53.4)
Diabetes	6,149 (19.5)	5,828 (24.7)	2,151 (15.9)
Cancer	4,583 (15.2)	4,977 (21.6)	1,880 (13.8)
**CVD types**			
CHD	8,456 (28.0)	9,305 (40.6)	3,763 (28.1)
Stroke	5,841 (18.6)	4,957 (20.2)	2,920 (20.6)
Heart attack	5,932 (19.2)	7,419 (31.9)	3,741 (28.0)
Angina	5,020 (15.9)	5,158 (22.2)	2,468 (18.9)
Other heart disease	17,754 (61.7)	12,454 (54.1)	7,174 (56.4)

*Values are n (%). CVD, cardiovascular disease; CHD, coronary heart disease; BMI, body mass index*.

### Association of Smoking Status With Mortality

**Figure 1:**
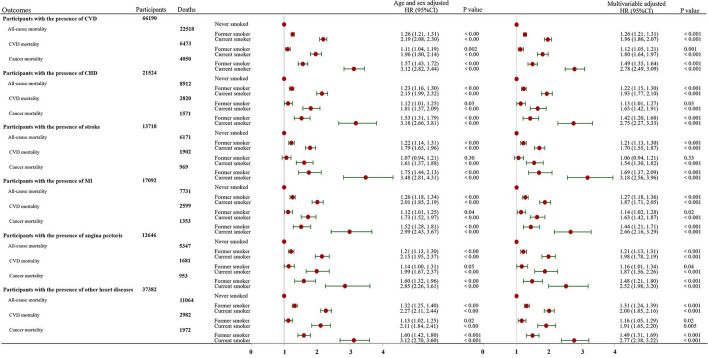
Association of smoking status with all-cause, CVD and cancer mortality stratified by the presence of cardiovascular disease. The multivariable-adjusted model was adjusted for age, sex, race, education, income, body mass index, physical activity, alcohol intake, baseline hypertension, diabetes and cancer variables. CI, confidence interval; HR, hazard ratio; CVD, cardiovascular disease; CHD, coronary heart disease; MI, myocardial infarction.

During a mean of 8.1 years of follow-up, a total of 22,518 deaths were recorded, including 6,473 CVD deaths, 4,050 cancer deaths and 11,995 other cause deaths. In the multivariable-adjusted model, current smokers and former smokers had a higher risk of all-cause, CVD and cancer mortality than never smokers (Figure 1). The corresponding HRs and 95% CIs among former smokers and current smokers were 1.26 (95% CI: 1.21 to 1.31) and 1.96 (95% CI: 1.86 to 2.07) for all-cause mortality, 1.12 (95% CI: 1.05 to 1.21) and 1.80 (95% CI: 1.64 to 1.97) for CVD mortality, and 1.49 (95% CI: 1.35 to 1.64) and 2.78 (95% CI: 2.49 to 3.09) for cancer mortality, respectively. Furthermore, similar results were found when the study subjects were stratified according to sex (Appendix Table 1). The results were also similar in patients with specific types of CVD, except CVD mortality among former smokers with the presence of stroke (HR, 1.06; 95% CI: 0.94 to 1.21) (Figure 1).

### Aassociation of Smoking Intensity With Mortality

**Figure 2:**
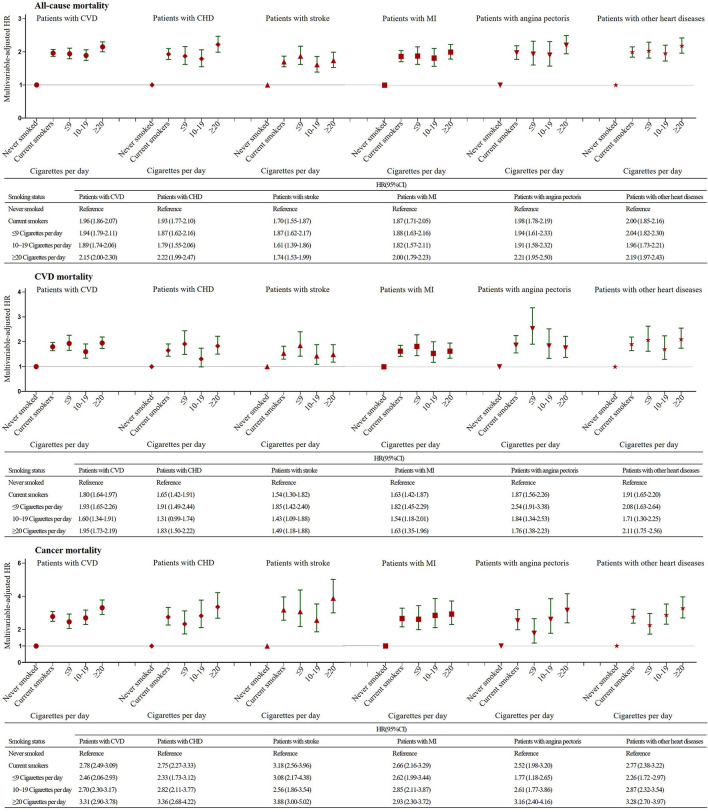
Association of Smoking Intensity with All-cause, CVD and Cancer Mortality Stratified by the Presence of Cardiovascular Disease. The multivariable-adjusted model was adjusted for age, sex, race, education, income, body mass index, physical activity, alcohol intake, baseline hypertension, diabetes and cancer variables. CI, confidence interval; HR, hazard ratio; CVD, cardiovascular disease; CHD, coronary heart disease; MI, myocardial infarction.

In comparison to never smokers, people who smoked ≤ 9, 10–19, and ≥20 cigarettes per day had a substantially higher risk of dying from all causes, CVD and cancer, regardless of the specific type of CVD (Figure 2). It is worth noting that there was a dose-dependent association between smoking intensity and cancer-related mortality in each CVD group. However, the risks of all-cause and CVD mortality did not increase in a monotonic fashion with an increasing number of cigarettes smoked per day in each CVD group (Figure 2). More interestingly, we found that risks decreased as the number of cigarettes smoked daily increased in the case of deaths from CVD in participants with angina pectoris and stroke but not in participants in other CVD groups (Figure 2).

### Association of the Number of Years Since Smoking Cessation With Mortality

**Figure 3:**
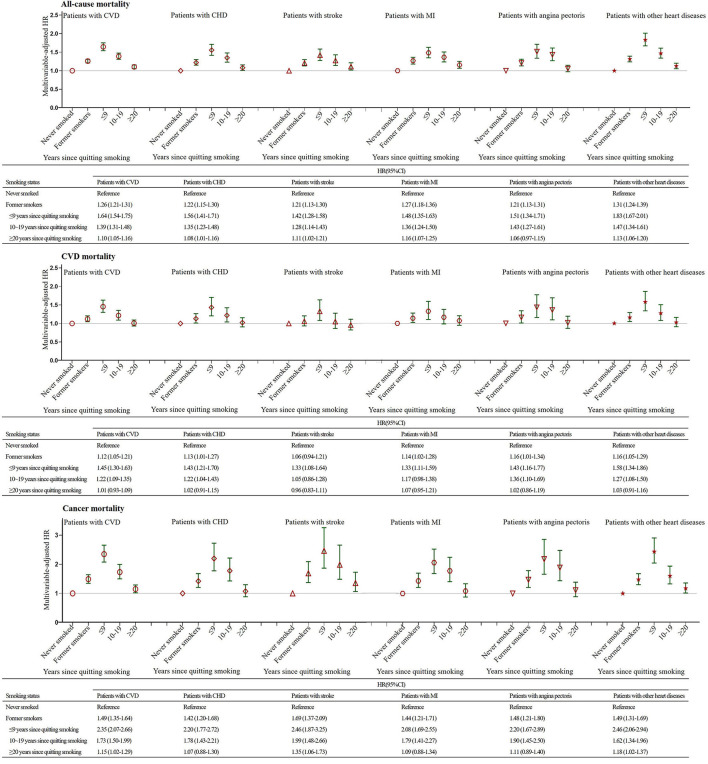
Association of the Number of Years Since Smoking Cessation with All-cause, CVD and Cancer Mortality Stratified by the Presence of Cardiovascular Disease. The multivariable-adjusted model was adjusted for age, sex, race, education, income, body mass index, physical activity, alcohol intake, baseline hypertension, diabetes and cancer variables. CI, confidence interval; HR, hazard ratio; CVD, cardiovascular disease; CHD, coronary heart disease; MI, myocardial infarction.

As reported in Figure 3, the risks for all-cause, CVD-related and cancer-related mortality decreased as the number of years since quitting increased in former smokers among all CVD groups. For smokers who had stopped smoking for ≥20 years, the risk of CVD mortality gradually approached the level of never smokers (Figure 3). Nevertheless, former smokers who had quit smoking ≥20 years prior did not have the same risk of all-cause mortality as never smokers in any CVD groups except the angina pectoris group (Figure 3). The cancer mortality risk for smokers with CVD, stroke and other heart disease who had stopped smoking for ≥20 years was also not identical to that of participants who had never smoked (Figure 3). Additionally, former smokers who had stopped smoking for ≥30 years had significant 45, 45, and 64% reductions in the risk of all-cause, CVD and cancer mortality, respectively, compared to continuing smokers in the group of CVD patients (Appendix Table 2).

Nevertheless, when never smokers were used as the reference group, we further observed that former smokers who quitted for ≥30 years still had higher all-cause mortality than never-smokers (Appendix Table 2). However, the differences in CVD mortality among never smokers and former smokers who quitted for ≥20 years, were not statistically significant (Appendix Table 2). Moreover, after 30 years since quitting, the risk of dying from cancer causes among former smokers were also at the same level of that observed among never smokers (Appendix Table 2). Therefore, the risk of dying from CVD and cancer causes returned similar to that of never smoker after 20 and 30 years since quitting, respectively. Yet, the risk of dying from all causes did not return similar to that of never smoker after 30 years since quitting.

### Association of the age of Smoking Initiation With Mortality

**Figure 4:**
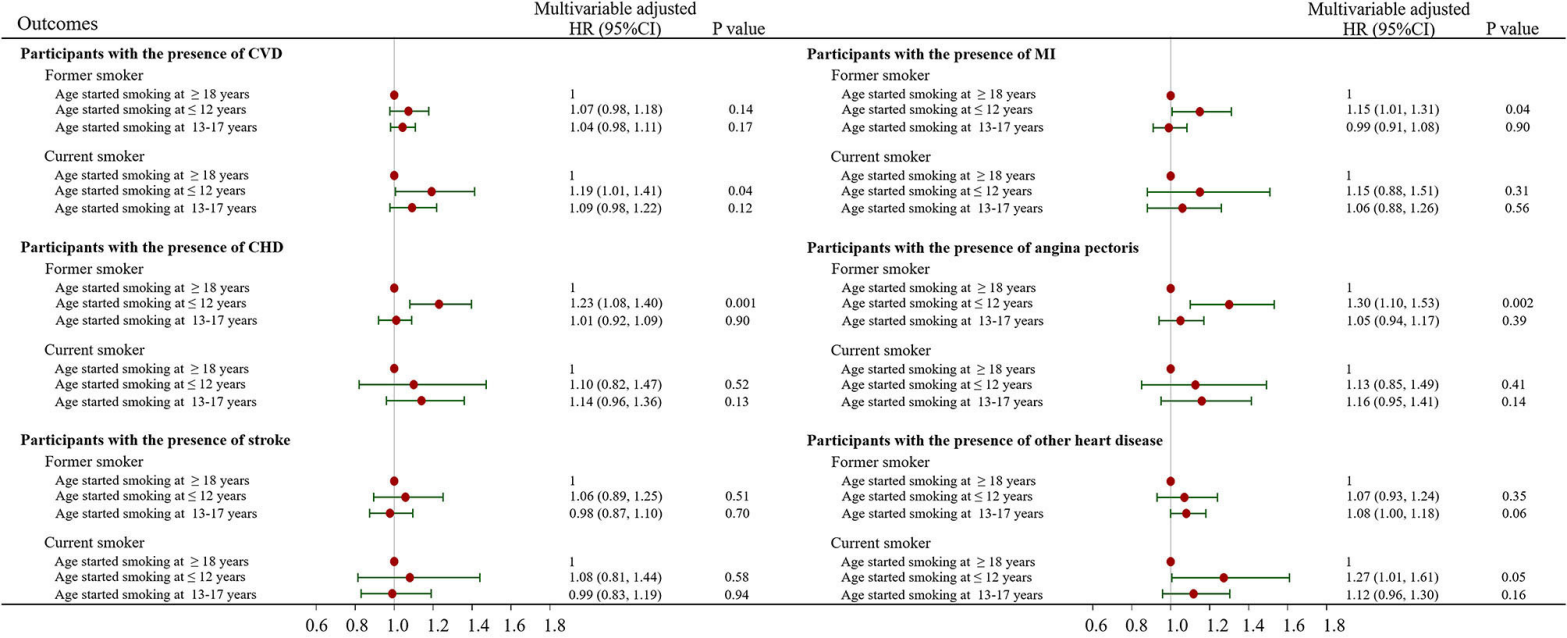
Association of the age of smoking initiation with all-cause, CVD and cancer mortality stratified by the presence of cardiovascular disease. The multivariable-adjusted model was adjusted for age, sex, race, education, income, body mass index, physical activity, alcohol intake, baseline hypertension, diabetes and cancer variables. CI, confidence interval; HR, hazard ratio; CVD, cardiovascular disease; CHD, coronary heart disease; MI, myocardial infarction.

For current smokers, all-cause mortality hazards were 19% higher and 27% higher for those who started smoking before the age of 12 years in the CVD group (HR, 1.19; 95% CI, 1.01 to 1.41) and in the other heart disease group (HR, 1.27; 95% CI, 1.01 to 1.61), respectively, but not among the remaining 4 CVD groups (Figure 4). For former smokers, in comparison to the age of smoking initiation in the 18+ years category, participants who started smoking at age 12 or younger than 12 years had an increased risk of all-cause mortality in the CHD, MI and angina pectoris groups (Figure 4). Compared with current and former smokers who started smoking at age 18 or older, current and former smokers who started smoking at age 13 to 17 years did not have different all-cause mortality regardless of the CVD group (Figure 4).

### Sensitivity Analysis

Excluding deaths that occurred during the first 2 years of follow-up or participants who had cancer at baseline did not substantially alter the significant associations of smoking status (never smokers, former smokers and current smokers) with all-cause or CVD mortality among patients with CVD (Appendix Table 3).

## Discussion

The present study showed that current and former smokers had a higher risk of all-cause, CVD and cancer mortality among patients with CVD, irrespective of the specific type of CVD. Among CVD patients, smokers had a higher risk for cancer mortality than for CVD mortality. Additionally, we found a dose-dependent positive association between smoking and cancer mortality, but the association for all-cause mortality was presented in a non-dose-response manner. Moreover, we observed that the risk of all-cause, CVD and cancer mortality declined as the number of years since quitting increased among patients with a previous CVD, and 20 or more than 20 years of smoking cessation ceased to be significantly related to increased mortality risk. Finally, early smoking initiation before 12 years of age was associated with increased risk for all-cause mortality among current smokers who had a history of CVD.

Numerous previous studies showed that smoking increased the risk of long-term mortality in patients with acute myocardial infarction by more than 19 to 43% (20, 26, 27). Similar results were also observed in patients who had been diagnosed with stable ischaemic heart disease (49%) (28), arterial disease (66%) (29), atrial fibrillation (82%) (30), and stroke (36%) (31). Moreover, our findings have also revealed that smokers had a 96% higher risk of long-term all-cause mortality than never smokers among patients with CVD. Obviously, these findings are much lower than those related to the contemporary risks of smoking from the general UK, US and Japan populations, where current smokers had a 180 to 200% excess risk of all-cause mortality (HR, 2.8 to 3.0) compared with the mortality of never smokers (21, 32, 33). The reasons for these inconsistencies between the CVD population and the general population are unclear, and further study is required.

Numerous prior studies tended to focus on the all-cause and CVD mortality effects of smoking on CVD patients. Our study improves upon prior long-term studies by exploring the differences in this association with smoking between various causes of death, especially cancer. Surprisingly, in the present study, former and current smokers had a higher HR for mortality due to cancer than for mortality due to CVD. Our findings are consistent with emerging evidence from the stroke population that showed that smoking increased the risk of CVD and cancer mortality, with HRs of 1.15 (95% CI, 0.88 to 1.50) and 3.83 (95% CI, 2.48 to 5.91) for CVD and cancer mortality, respectively (31). Several explanations may account for the findings that smoking in CVD patients has a greater impact on cancer mortality than on CVD mortality. First, some socioeconomic and clinical factors might attenuate the association between smoking and death from CVD (31). Another explanation is that smokers with CVD might undergo more aggressive CVD treatments and secondary prevention, which reduce their risk of CVD mortality. In addition, smoking potentiates responsiveness to clinical treatments, particularly antiplatelet therapies, in the CVD population (34–37).

Our analysis showed a significant positive dose-response association between smoking intensity and cancer mortality but not all-cause or CVD mortality. Inconsistent with our findings, previous studies showed that the risk of all-cause CVD and cancer mortality increased significantly with an increasing number of cigarettes smoked per day (13, 14, 32). Furthermore, we found a more interesting result that the risk of death from CVD decreased significantly with smoking intensity in stroke and angina patients. A possible biological mechanism to explain this phenomenon might be an ingestion of increased amounts of nicotine with smoking intensity that might induce P2Y12 receptor expression in human platelet lysates (within the dose range) (37), which may increase the relative benefit of antiplatelet therapy in smokers compared to that of non-smokers. However, the exact underlying mechanisms remain to be determined.

It turns out that smoking cessation has multiple benefits for different populations, and the CVD population is no exception (5, 13, 15, 21, 29, 31, 32, 38, 39). Previous studies showed that smokers who ceased smoking at 25 to 34, 35 to 44, or 45 to 54 years of age gained approximately 10, 9, and 6 years of life, respectively, relative to persistent smokers (21). This result revealed the beneficial effect of smoking cessation on mortality. In agreement with previous studies, our results also confirm that the risk of death from all causes, CVD and cancer substantially declined with an increasing number of years since quitting, with risks equivalent to those of never smokers after 30 years of cessation. These findings, the absolute benefits of quitting smoking, suggest that further work is needed to encourage smoking cessation in patients with CVD. Contemporary guidelines also emphasize that cigarette/tobacco cessation is an important treatment for primary and secondary CVD prevention and is the most cost-effective strategy for CVD prevention (40, 41). Although the benefits of smoking cessation among patients with CVD are well established in this study and other previous studies, the true gain of life-years from the time of cessation in the CVD population remains unclear. Therefore, future studies should focus on the quantitative relationship between the time of smoking cessation and the true gain of life-years in the CVD population.

Numerous prior studies have assessed the relationship between the age of smoking initiation and smoking-related morbidities and mortality in the general population (32, 42–45), and most studies have shown a strong inverse association of mortality with age at initiation (32, 42–45). However, data on the impact of the age at smoking initiation on adverse outcomes in CVD patients are weak and unclear. Although our study showed that a young age at smoking initiation was associated with increased risk, the differences were not statistically significant. It is likely that the intensity and duration of smoking may have disturbed the relationship between the age of smoking initiation and mortality.

## Limitations

The study has several potential limitations. First, although our results remained robust after a series of sensitivity analyses, we cannot establish a cause-effect relationship between smoking and mortality in CVD patients due to the observational nature of our study. Second, smoking status was collected with the use of self-reported responses only at baseline, and it is possible that smoking status is subject to measurement error and might have changed throughout follow-up. Third, this study did not collect information on what types of tobacco products were smoked, and the potential influence of types of tobacco products could not be tested, which should be evaluated in future studies. Fourth, although we were able to adjust for many confounding factors in our study, residual and unmeasured confounding factors cannot be fully ruled out. For example, blood pressure, blood glucose, lipid profiles (Low Density Lipoprotein-Cholesterol, High Density Lipoprotein-Cholesterol, Tryglicerides) and treatments are some of the factors that can clearly bias the relationship between smoke and mortality outcomes. Therefore, the true strength of association remains uncertain. Fifth, this study did not adjust for multiple comparison. In addition, there were overlaps in the subgroups of CVD, which might make it harder to draw conclusions about the impact of smoking status on the risk of all-cause, CVD and cancer mortality in the specific CVD subgroup. Therefore, future studies should focus on examining the effect of smoking status on the risk of death from all-cause, CVD and cancer in patients with a specific CVD disease. Finally, since the NHIS is a passive follow-up study that relies on probabilistic matching to the NDI to assess the death of participants, the cause of death may be misclassified and the estimated hazards of cause-specific mortality, but not the estimated hazards of all-cause mortality, may have been affected.

In conclusion, the findings of this prospective cohort study using a large sample of U.S. adults with CVD suggest that there is a higher all-cause, CVD and cancer mortality among former and current smokers than never smokers but did not reveal a dose-response pattern with respect to smoking intensity in current smokers. In addition, an inverse association between the age of smoking initiation and mortality was not observed. This by no means indicates that it should not be encouraged to stop or reduce smoking among patients with CVD or to control tobacco use among youth. We further found that the risk of all-cause, CVD and cancer mortality declined significantly with increasing time since cessation of smoking, which suggests that smoking is an important modifiable risk factor for mortality in the CVD population.

## Data Availability Statement

Publicly available datasets were analyzed in this study. This data can be found here: https://www.cdc.gov/nchs/nhis/index.htm.

## Ethics Statement

The studies involving human participants were reviewed and approved by in this study, only public-use NHIS data were used. The public-use NHIS data are de-identified and do not include any protected health information. Our study was based on secondary analysis of public-use data. The publicly available data are considered as exempt under the ethical board review of the corresponding author's institution (The Third Xiangya Hospital of Central South University). The patients/participants provided their written informed consent to participate in this study.

## Author Contributions

J-lW and X-cZ: conception and design of the study and administrative support. J-lW, Y-fW, and X-cZ: data analysis and interpretation. J-lW, W-jY, L-yZ, and X-cZ: original draft preparation. All authors final approval of manuscript. All authors contributed to the article and approved the submitted version.

## Funding

This study was funded by the National Natural Science Foundation of China (nos. 81773822 and 81973400).

## Conflict of Interest

The authors declare that the research was conducted in the absence of any commercial or financial relationships that could be construed as a potential conflict of interest.

## Publisher's Note

All claims expressed in this article are solely those of the authors and do not necessarily represent those of their affiliated organizations, or those of the publisher, the editors and the reviewers. Any product that may be evaluated in this article, or claim that may be made by its manufacturer, is not guaranteed or endorsed by the publisher.
