# Supplementary material for: Association Between Initiation, Intensity, and Cessation of Smoking and Mortality Risk in Patients With Cardiovascular Disease: A Cohort Study
Source: Front Cardiovasc Med. 2021 Dec 15;8:728217. doi: 10.3389/fcvm.2021.728217 (PMC8714779; doi:10.3389/fcvm.2021.728217)
Supplement: Supplementary file 1 [file Data_Sheet_1.docx]

**Appendix table 1. Hazard ratios of all-cause, CVD and cancer mortality, according to smoking status and sex.**

| **Sex** | | **Women** | | | | | | **Men** | | | |
| --- | --- | --- | --- | --- | --- | --- | --- | --- | --- | --- | --- |
| Mortality and smoking status | Model 1† | | | Model 2‡ | | | Model 1† | | | Model 2‡ | |
|  | HR (95%CI) | | P value | HR (95%CI) | P value | HR (95%CI) | | | P value | HR (95%CI) | P value |
| All-cause mortality |  | |  |  |  |  | | |  |  |  |
| Never smoked | Reference | |  | Reference |  | Reference | | |  | Reference |  |
| Former smoker | 1.30 (1.24-1.36) | | <0.001 | 1.33 (1.26-1.40) | <0.001 | 1.25 (1.18-1.32) | | | <0.001 | 1.21 (1.14-1.28) | <0.001 |
| Current smoker | 2.13 (2.00-2.28) | | <0.001 | 2.02 (1.88 -2.18) | <0.001 | 2.22 (2.08-2.38) | | | <0.001 | 1.90 (1.76 -2.04) | <0.001 |
| CVD mortality |  | |  |  |  |  | | |  |  |  |
| Never smoked | Reference | |  | Reference |  | Reference | | |  | Reference |  |
| Former smoker | 1.10 (1.00-1.21) | | 0.05 | 1.12 (1.05-1.21) | 0.001 | 1.17 (1.06-1.29) | | | 0.002 | 1.15 (1.04-1.27) | <0.001 |
| Current smoker | 1.87 (1.63-2.14) | | <0.001 | 1.80 (1.64-1.97) | <0.001 | 2.07 (1.83-2.33) | | | 0.01 | 1.78 (1.54-2.06) | <0.001 |
| Cancer mortality |  | |  |  |  |  | | |  |  |  |
| Never smoked | Reference | |  | Reference |  | Reference | | |  | Reference |  |
| Former smoker | 1.70 (1.50-1.92) | | <0.001 | 1.65 (1.43-1.91) | <0.001 | 1.45 (1.28-1.64) | | | <0.001 | 1.33 (1.17-1.52) | <0.001 |
| Current smoker | 3.11 (2.66-3.64) | | <0.001 | 2.98 (2.51-3.54) | <0.001 | 3.06 (2.66-3.51) | | | <0.001 | 2.59 (2.25-2.98) | <0.001 |

†Model 1 was adjusted for age and sex.

‡Model 2 was additionally adjusted as follows: body mass index, race or ethnicity, educational attainment, household income, alcohol intake, physical activity and self-reported physician-diagnosis of disease.

HR = hazard ratio; CI = confidence interval; CVD = cardiovascular disease.

**Appendix table 2. Risk associated with** **years since quitting in former smokers compared to current smokers or never smokers for all-cause, CVD and cancer mortality**

| Mortality and smoking status | All-cause mortality | | | CVD mortality | | Cancer mortality | | |
| --- | --- | --- | --- | --- | --- | --- | --- | --- |
|  | HR (95%CI) | P value | HR (95%CI) | | P value | | HR (95%CI) | P value |
| Current smokers | Reference |  | Reference | |  | | Reference |  |
| Former smokers |  |  |  | |  | |  |  |
| ≤9 years since quitting | 0.81 (0.76-0.87) | <0.001 | 0.80 (0.70-0.91) | | <0.001 | | 0.80 (0.71-0.90) | <0.001 |
| 10-19 years since quitting | 0.68 (0.63-0.73) | <0.001 | 0.66 (0.58-0.75) | | <0.001 | | 0.57 (0.50-0.66) | <0.001 |
| 20-29 years since quitting | 0.56 (0.51-0.61) | <0.001 | 0.57 (0.49-0.66) | | <0.001 | | 0.47 (0.39-0.56) | <0.001 |
| ≥30 years since quitting | 0.55 (0.52-0.58) | <0.001 | 0.55 (0.49-0.61) | | <0.001 | | 0.36 (0.32-0.41) | <0.001 |
| Never smokers | 0.50 (0.47-0.52) | <0.001 | 0.54 (0.49-0.60) | | <0.001 | | 0.34 (0.30-0.38) | <0.001 |
| Never smokers | Reference |  | Reference | |  | | Reference |  |
| Former smokers |  |  |  | |  | |  |  |
| ≤9 years since quitting | 1.64 (1.54-1.74) | <0.001 | 1.45 (1.30-1.63) | | <0.001 | | 2.34 (2.07-2.63) | <0.001 |
| 10-19 years since quitting | 1.39 (1.30-1.47) | <0.001 | 1.22 (1.09-1.36) | | <0.001 | | 1.72 (1.50-1.98) | <0.001 |
| 20-29 years since quitting | 1.12 (1.04-1.22) | 0.005 | 1.04 (0.91-1.20) | | 0.54 | | 1.40 (1.17-1.67) | <0.001 |
| ≥30 years since quitting | 1.10 (1.04-1.15) | <0.001 | 1.00 (0.92-1.09) | | 0.99 | | 1.07 (0.95-1.22) | 0.28 |
| Current smokers | 2.01 (1.90-2.12) | <0.001 | 1.84 (1.67-2.02) | | <0.001 | | 2.91 (2.61-3.25) | <0.001 |

Note: The reference category group is current smoker or never smoker. HR, hazard ratio; CI, confidence interval; CVD, cardiovascular disease.

**Appendix table 3. Sensitivity analysis of the association of smoking status with all-cause, CVD and cancer mortality.**

| Employment status | Participants | Deaths | Never smoked | | | Former smoker | | Current smoker | |
| --- | --- | --- | --- | --- | --- | --- | --- | --- | --- |
|  |  |  | Reference | | HR (95%CI) | | P value | HR (95%CI) | P value |
|  |  |  |  |  | | |  |  |  |
| All-cause mortality |  |  |  |  | | |  |  |  |
| Persons who died in the first 2 years of follow up were excluded | 61503 | 17831 | 1 | 1.24 (1.19-1.30) | | | <0.001 | 2.05 (1.93-2.17) | <0.001 |
| Persons with CVD or cancer at baseline were excluded | 54687 | 17309 | 1 | 1.25 (1.19-1.31) | | | <0.001 | 1.99 (1.88-2.12) | <0.001 |
| CVD mortality |  |  |  |  | | |  |  |  |
| Persons who died in the first 2 years of follow up were excluded | 61503 | 5019 | 1 | 1.12 (1.03-1.21) | | | 0.01 | 1.85 (1.66-2.06) | <0.001 |
| Persons with CVD or cancer at baseline were excluded | 54687 | 5230 | 1 | 1.16 (1.08-1.26) | | | <0.001 | 1.80 (1.62-2.01) | <0.001 |
| Cancer mortality |  |  |  |  | | |  |  |  |
| Persons who died in the first 2 years of follow up were excluded | 61503 | 3097 | 1 | 1.48 (1.33-1.66) | | | <0.001 | 2.97 (2.63-3.35) | <0.001 |
| Persons with CVD or cancer at baseline were excluded | 54687 | 2629 | 1 | 1.57 (1.38-1.78) | | | <0.001 | 3.35 (2.94-3.82) | <0.001 |

CI = confidence interval; HR = hazard ratio; CVD = cardiovascular disease.
